# Clear Cell Adenocarcinoma of the Ureter Similar to Clear Cell Renal Cell Carcinoma Histology

**DOI:** 10.1155/2021/5599014

**Published:** 2021-05-28

**Authors:** Kazuhiro Watanabe, Go Hasegawa, Yohei Ikeda, Noboru Hara, Tsutomu Nishiyama

**Affiliations:** ^1^Department of Urology, Uonuma Institute of Community Medicine, Niigata University Medical and Dental Hospital, Urasa 4132, Minamiuonumashi, Niigata 949-7302, Japan; ^2^Department of Pathology, Uonuma Institute of Community Medicine, Niigata University Medical and Dental Hospital, Minamiuonumashi, Niigata 949-7302, Japan; ^3^Department of Diagnostic Radiology, Uonuma Institute of Community Medicine, Niigata University Medical and Dental Hospital, Minamiuonuma, Niigata, Japan

## Abstract

A 70-year-old woman was referred to our hospital with gross hematuria and diagnosed with right invasive ureteral cancer and bladder urothelial carcinoma in situ. Intravesical BCG therapy and neoadjuvant chemotherapy with carboplatin and gemcitabine were performed at the same time. Subsequently, laparoscopic right nephroureterectomy was performed. Urothelial carcinoma in situ persisted; however, most of the tumor was clear cell carcinoma. The clear cell carcinoma lesion had clear cytoplasm with round nuclei and visible nucleoli in an insular arrangement as is the case with clear cell renal cell carcinoma. No transitional lesion between clear cell adenocarcinoma and urothelial carcinoma was presented. The clear cell carcinoma lesion was GATA3 negative and HNF4*α* positive; however, the urothelial cancer lesion was GATA3 positive and HNF4*α* negative. Clear cell carcinoma was diagnosed as clear cell adenocarcinoma similar to clear cell renal cell carcinoma histology.

## 1. Introduction

Clear cell adenocarcinoma of the urinary tract, that is a subtype of tumors of Müllerian type, is a rare genitourinary malignancy occurring predominantly in the bladder or urethra; however, it rarely occurs in the ureter [1]. Clear cell renal cell carcinoma is the most common histology of renal cell carcinoma. However, metastases to the urinary tract are extremely rare. Furthermore, clear cell adenocarcinoma of the ureter similar to clear cell renal cell carcinomas in an extrarenal location is extremely rare.

## 2. Case Report

**Figure 1:**
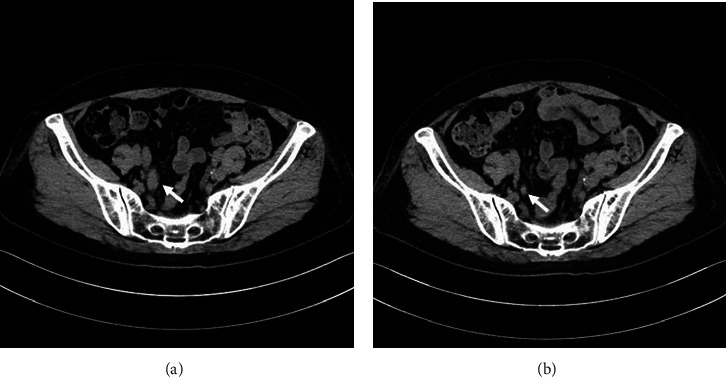
CT findings. (a) Plain CT at diagnosis shows a right invasive ureteral tumor and no metastatic findings ((a) arrow). (b) Plain CT after four course of chemotherapy shows the right ureteral tumor diminished in size after the therapy ((b) arrow).

**Figure 2:**
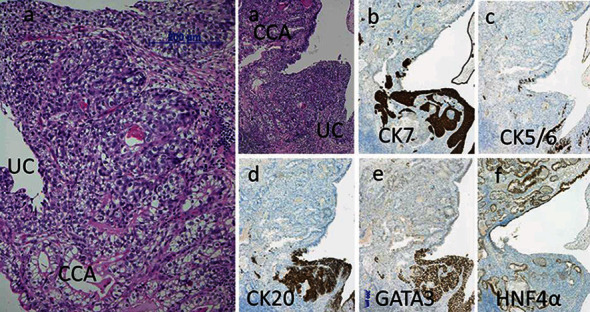
Pathological findings. Histopathological findings reveal that urothelial carcinoma in situ ((a) UC) persisted in part; however, most of the tumor is clear cell adenocarcinoma ((a) CCA) ((a) hematoxylin and eosin). The clear cell adenocarcinoma lesion has clear cytoplasm with round nuclei and visible nucleoli in an insular arrangement. No transitional lesion between clear cell adenocarcinoma and urothelial carcinoma can be presented in this lesion. The clear cell adenocarcinoma lesion is CK7 negative (b), CK5/6 negative (c), CK20 negative (d), GATA3 negative (e), and HNF4*α* positive (f). The urothelial cancer lesion is CK7 positive (b), CK5/6 weak positive (c), CK20 positive (d), GATA3 positive (e), and HNF4*α* negative (f). CCA: clear cell adenocarcinoma; UC: urothelial carcinoma.

In December 2019, a 70-year-old woman was referred to our hospital with awareness of gross hematuria. Abdominal ultrasonography showed dilatation of the right renal pelvis and calyces. Cystoscopy showed a reddish abnormal mucosa including papillary tumors on the posterior and dome of the bladder wall. Because of her severe renal function, she could not use the administration of contrast media while computed tomography (CT) examination. Plain CT showed a right invasive ureteral tumor and no renal tumors and no metastatic findings (Figure 1(a)). Urine cytology was class V in five classes (negative, suspicious, and positive corresponding to classes I–II, III, and IV–V, respectively). In January 2020, transurethral resection of the bladder tumor (TUR-BT) was performed. Histopathological findings were urothelial carcinoma in situ. The patient was diagnosed with right invasive ureteral cancer and bladder urothelial carcinoma in situ. We made a treatment proposal to the patient: intravesical Bacillus Calmette-Guerin (BCG) therapy for preservation of urinary bladder and neoadjuvant chemotherapy for right invasive ureteral cancer, thereafter laparoscopic right nephroureterectomy. The patient was consented for the treatment. Intravesical BCG therapy, 80 mg/40 ml normal saline solution, once a week, was performed 8 times. She could not use cisplatin for standard anticancer drug because of her severe renal function. Creatinine clearance (eGFR) was determined by using serum creatinine and the Cockcroft and Gault formula. Her eGFR was 20.9 ml/min/1.73 m^2^. Four courses of neoadjuvant chemotherapy with carboplatin instead of cisplatin and gemcitabine were performed at the same time of BCG therapy. The patient was design to be treated on an outpatient basis. Gemcitabine 1100 mg/body was design to be given by intravenous infusion over 30 minutes on day 1, 8 and 15 of a 28-day cycle; however, day 15, gemcitabine was omitted because of thrombopenia. Carboplatin dosed to an AUC of 5 (250 mg/body) was given as an intravenous infusion over one hour on day 1 of a 28-day cycle. The cancer cells in urine were disappeared, and the right ureteral tumor diminished in size after the therapy (Figure 1(b)). In May 2020, laparoscopic right nephroureterectomy was performed. Histopathological findings of the lesion revealed that urothelial carcinoma in situ persisted in part; however, most of the tumor was clear cell carcinoma similar to clear-cell renal cell carcinomas, pT1, and margin negative. The clear cell carcinoma lesion of the present case had clear cytoplasm with round nuclei and visible nucleoli in an insular arrangement as is the case with clear cell renal cell carcinoma. The clear cell carcinoma lesion was CK5/6 negative, CK20 negative, GATA3 negative, CK7 negative, 34*β*-E12 negative, CD10 positive, EMA weak positive, vimentin negative, HNF4*α* positive, Uroplakin negative, PAX8 negative, and CEA weak positive; however, the urothelial cancer lesion was CK7 positive, CK5/6 weak positive, CK20 positive, GATA3 positive, 34*β*-E12 negative, CD10 weak positive, EMA weak positive, vimentin negative, HNF4*α* negative, Uroplakin weak positive, PAX8 weak positive, and CEA negative (Table 1, Figure 2). No transitional lesion between clear cell carcinoma and urothelial carcinoma was presented in the ureteral lesion in the present case. HNF1B was negative for both clear cell adenocarcinoma lesion and urothelial cancer lesion. Therefore, clear cell carcinoma in the present case was diagnosed with clear cell adenocarcinoma similar to clear cell renal cell carcinoma histology rather than clear cell urothelial carcinoma of a variant of urothelial carcinoma. No clear cell renal cell carcinoma lesion was found in the kidney of the nephroureterectomy specimen. For these reasons, the pathological diagnosis of the present case was clear cell adenocarcinoma of the ureter similar to clear cell renal cell carcinoma histology.

The patient is being followed up for nine months without postoperative adjuvant therapy and without tumor recurrence.

## 3. Discussion

Clear cell carcinoma that occurs in the urinary tract is rare and generally clear cell urothelial carcinoma or clear cell carcinoma of the Müllerian type [ 1]. Renal-type clear cell carcinoma occurring as a primary tumor in an extrarenal location is extremely rare, and there are several case reports of renal-type clear cell carcinoma occurring in the prostate [2]. We described a case of a clear cell adenocarcinoma of the ureter with the histologic and immunohistochemical features identical to that of clear cell carcinoma occurring in the kidney. In 2016, the *WHO Classification of Tumours of the Urinary System and Male Genital Organs* was revised [1]. With this revision, clear cell adenocarcinoma is classified as tumor of Müllerian type. Urinary tract clear cell adenocarcinoma occurs primarily in women and is thought to originate from Müllerian progenitor cells present in the bladder wall or adjacent soft tissues. On the other hand, reported cases of clear cell urothelial carcinoma were male [3]. Müllerian type clear cell adenocarcinoma is said to be immunoreactive to PAX8, HNF1B, CA125, and p53 [ 1]. On the other hand, clear cell urothelial carcinoma is a variant of urothelial carcinoma and is said to show positive findings of CK5/CD44, CK20, PAX8, and GATA3/CK7 [1]. In the present case, histopathological findings revealed that urothelial carcinoma in situ persisted in part; however, most of the tumor was clear cell adenocarcinoma, pT1, and margin negative. The clear cell adenocarcinoma lesion of the present case had clear cytoplasm with round nuclei and visible nucleoli in an insular arrangement as is the case with clear cell renal cell carcinoma. The clear cell adenocarcinoma lesion of the present case was CK5/6 negative, CK20 negative, GATA3 negative, CK7 negative, and HNF4*α* positive; however, the urothelial cancer lesion was CK5/6 weak positive, CK20 positive, GATA3 positive, and HNF4*α* negative (Figure 2). Müllerian type clear cell adenocarcinomas should be characterized by the usual tubulocystic, papillary, or diffuse growth patterns and should have hobnail cells with scant cytoplasm and hyperchromatic dense nuclei but were not observed in the present case [1]. No transitional lesion between clear cell adenocarcinoma and urothelial carcinoma was presented in the ureteral lesion in the present case. Immunohistochemically in the present case, HNF1B classified as tumor of Müllerian type was negative for both clear cell adenocarcinoma lesion and urothelial cancer lesion; however, HNF4*α* was positive for clear cell adenocarcinoma lesion similar to clear cell renal cell carcinomas [4]. Based on the above results, clear cell carcinoma in the present case was diagnosed as clear cell adenocarcinoma similar to clear cell renal cell carcinoma histology. Clear cell carcinomas that occur in the urinary tract have been reported in various articles; however, a clear cell adenocarcinoma of the ureter similar to clear cell renal cell carcinoma histology has not been confirmed in the reported articles.

## 4. Conclusion

We reported an extremely rare clear cell adenocarcinoma of the ureter that showed histology similar to clear cell renal cell carcinomas other than urothelial carcinoma variant or clear cell carcinoma of the Müllerian type.

## Figures and Tables

**(a):** 

	CK7	CK20	CK5/6	Uroplakin	GATA3	CD10	34*β*-E12
UC	(+)	(+)	(+/-)	(+/-)	(+)	(+/-)	(-)
CCC	(-)	(-)	(-)	(-)	(-)	(+)	(-)

**(b):** 

	EMA	Vimentin	PAX8	CEA	HNF4*α*	HNF1*β*	
UC	(+/-)	(-)	(+/-)	(-)	(-)	(-)	
CCC	(+/-)	(-)	(-)	(+/-)	(+)	(-)	

UC: urothelial carcinoma; CCC: clear cell adenocarcinoma; (+): positive; (+/-): weak positive; (-): negative.
